# Influence of Carboxymethyl Cellulose on the Stability, Rheological Property, and *in-vitro* Digestion of Soy Protein Isolate (SPI)-Stabilized Rice Bran Oil Emulsion

**DOI:** 10.3389/fnut.2022.878725

**Published:** 2022-04-11

**Authors:** Wenguan Zhang, Jia Hao, Yanan Yuan, Duoxia Xu

**Affiliations:** Beijing Key Laboratory of Flavor Chemistry, Beijing Laboratory for Food Quality and Safety, Beijing Advanced Innovation Center for Food Nutrition and Human Health (BTBU), Beijing Engineering and Technology Research Center of Food Additives, School of Food and Health, Beijing Higher Institution Engineering Research Center of Food Additives and Ingredients, Beijing Technology and Business University, Beijing, China

**Keywords:** rice bran oil (RBO), emulsion, carboxymethyl cellulose (CMC), physicochemical stability, rheological properties, *in vitro* digestion

## Abstract

In this study, carboxymethyl cellulose (CMC) was added to soybean protein isolate (SPI)-stabilized rice bran oil (RBO) emulsion to improve its physicochemical stability and free fatty acid (FFA) release characteristics. RBO emulsions stabilized by SPI and various contents of CMC were prepared and assessed by measuring zeta potential, particle size, transmission, and microstructure, the rheological properties were analyzed by dynamic shear rheometer. In addition, its chemical stability was characterized by a storage experiment, and the FFA release was explored by a simulated gastrointestinal tract (GIT) model. It showed that the negative charge of the droplets of RBO emulsion was increased with increasing CMC content. The decrease in transmission of SPI-stabilized RBO emulsion with increasing CMC content was due to the droplets not being free to move by the special network interaction and an increase in the viscosity. According to the determination of the reactive substances of lipid hydroperoxide and thiobarbituric acid during 30 days storage at 37°C, the chemical stability of the emulsion added with CMC was enhanced compared with the SPI-stabilized RBO emulsion. *In-vitro* digestion studies not only evaluated the structural changes of RBO emulsions at different stages, but also found that RBO emulsion with CMC showed a higher level of free fatty acids release in comparison with that without CMC. It indicated that the utilization of CMC can improve the bioavailability of RBO emulsions.

## Introduction

In the recent years, rice bran oil (RBO) is now widely used in food processing. It suggests that RBO can promote human health, reduce human serum cholesterol, regulate human brain function, and may have certain preventive and therapeutic effects on vascular headaches and autonomic nervous dysfunction (1, 2). RBO with high content of unsaturated fatty acids in which the ratio of linoleic acid to oleic acid is close to 1:1 (3). It also contains a range of natural antioxidants, namely, vitamin E, oryzanol, squalene, active lipase, sitosterol, and other functional components (4–6). Compared with other vegetable oils, RBO has a relatively long shelf life due to high levels of natural antioxidants (7).

Therefore, there is interest in developing an RBO delivery system to benefit from its ideal nutritional and functional characteristics. RBO is highly lipid-soluble and can be incorporated into foods in the form of oil-in-water emulsions as an oil phase. However, the studies on RBO were mainly focused on refining, detection of active substances, and improvement of production process and storage methods, while the research on its delivery system and *in-vitro* digestion characteristics was limited. Studies have shown that the addition of surfactants (Twain 80 and Span80 mixture) can improve the oxidation stability of oil in RBO oil phase emulsion (8). The effect of natural emulsifier type on the stability of RBO emulsions found that the emulsion formed by a polysaccharide (modified starch and Arabia gum) has better thermal stability, salt stability, and pH stability than that of whey protein isolate emulsion. This may be due to the difference in colloidal interaction between oil droplets (9). Moreover, it was also reported that RBO-in-water pickering emulsions prepared by cellulose nanocrystals as stabilizers, gum Arabia and nonionic surfactant as emulsifiers showed gel-like behavior and high oxidative stability (10).

Soybean protein isolate (SPI), as the most important nutritional ingredient in soybean, is a high-quality vegetable protein of edible origin and contains eight essential amino acids required by the human body (11). It also has many important functional properties such as emulsifying, solubility, and gelation (12–14). These properties can significantly reduce the interfacial tension of oil-water or air-water further improve food functional properties, such as improving taste and storage property, increasing elasticity, water retention, and oil absorption. When it is used as an emulsifier in emulsion, it is reported that the protein only forms a thin interface layer around the lipid droplet. Therefore, the main stabilization mechanism is electrostatic exclusion. However, when repulsive electrostatic interaction decreases or attractive hydrophobic interaction increases, it tends to unstable aggregation. It is said that the stability mechanism of the protein-polysaccharide system is a steric hindrance and electrostatic repulsion. After adding polysaccharides, a thick interface layer can be formed around the droplet (15). Therefore, protein-polysaccharide encapsulated lipid droplets are more stable to changes in environmental conditions than protein encapsulated lipid droplets (16).

Carboxymethyl cellulose (CMC) is an anionic linear polymer. The Food and Agriculture Organization (FAO) and the WHO have approved the use of pure CMC in food as a good emulsion stabilizer and thickener, which can maintain the stability of food quality, prevent oil-water stratification, and prolong storage time. Previous studies had applied it to the production of ice cream and found that CMC could increase the emulsifying ability of protein and reduce fat agglomeration, which played a certain role in the homogenization of ice cream liquid (17, 18). Recently, CMC was reported as an oral delivery system. In contrast to all other polysaccharides, CMC has a hydrophilic character because of its carboxylate groups. These carboxylate groups are responsible for *in-situ* gelations, bioadhesion, sensitivity to environmental stimuli, and controlled drug release (19). Studies have shown that the addition of CMC into SPI-stabilized emulsion can effectively reduce the release of VD3 in the simulated *in-vitro* digestion process due to the cross-linked by CMC and Ca^2+^ had polysaccharide protection (20).

Therefore, the purpose of this study is to investigate the effects of different concentrations of CMC on the physicochemical stability and *in-vitro* digestion characteristics of SPI-stabilized RBO emulsion. The RBO emulsion was assessed by zeta potential, particle size, transmission, microstructure, and rheological behavior. The microstructural changes of RBO emulsions at different phases of *in-vitro* digestion and the FFA release in the small intestine stage were also evaluated. Ultimately, our goal is to apply plant protein-polysaccharide to improve the physicochemical stability and digestion capacity of RBO and extend the application of RBO in the food industry.

## Materials and Methods

### Materials

Soy protein isolate (SPI) was obtained from Shanghai YUANYE Biotechnology Corporation Ltd. (Shanghai, China). CMC was purchased from Changshu WEALTHY Technology Corporation Ltd. (Changshu, China). Rice bran oil (RBO) was purchased from Jinlongyu (Qinhuangdao, China). Mucin from the porcine stomach (CAS#84082-64-4, BR), pepsin from porcine gastric mucosa (CAS#9001-75-6, 10,000 U/g), bile salt from pigs (CAS#8008-63-7, more than 60% cholic acid content), and lipase from porcine pancreas (CAS#9001-62-1, BR, 30,000 U/g) were purchased from Shanghai YUANYE Biotechnology Corporation Ltd. (Shanghai, China). All other chemicals were of analytical grade.

### Preparation of Emulsions

Soy protein isolate (SPI) (1.5 wt%) and CMC (3 wt%) were first dissolved in the 10.0 mM phosphate buffer at pH 6.0, 0.02% sodium azide was added to prevent microbial growth. The solutions were kept overnight to ensure complete dissolution and dispersion. Then, RBO (10 wt%) was added to the SPI solution by using an Ultra-Turrax (B25 model, Shanghai Beierte experimental Equipment Co., Ltd) to prepare crude emulsion at 19,000 rpm for 3 min, which was subsequently homogenized using a microfluidizer processor (M-110PS model, Microfluidics International Corporation, Newton, MA) at 50 MPa for three times. Then, the SPI-RBO emulsion and CMC were homogenized with the ratio of 2:1 by using an Ultra-Turrax (B25 model, Shanghai Beierte experimental Equipment Corporation Ltd.) at 19,000 rpm for 3 min. Finally, the pH was adjusted to pH 3.5 using 1.0 M HCl or NaOH. Thus, final emulsions contained 1 wt% SPI, 6.67 wt% rice bran oil and various contents of CMC (0–0.625 wt%), respectively.

### Zeta Potential and Particle Size Measurements

Zeta potential and particle size of the samples were analyzed according to the method of Xu et al. (21). The zeta potential of SPI-RBO emulsions stabilized with different concentrations of CMC was determined by Zetasizer Nano-ZS90 (Malvern Instruments, Worcestershire, UK). Emulsions were diluted (1:400) with buffer solution (pH 3.5) before analysis to minimize multiple scattering effects. The data were collected over 11 continuous readings after the samples were equilibrated at 25°C for about 120 s.

To determine the average droplet size of emulsions, diluted samples (1:400) were loaded into the instrument at a fixed detector angle of 90°. Droplet size was described as cumulative mean diameter (size, μm).

### Physical Stability Measurements

The physical stability of SPI-RBO emulsions stabilized with different concentrations of CMC was measured by using LUMiSizer (LUM GmbH, Berlin, Germany). The instrumental parameters of the measurement were as follows: volume, 0.4 ml of dispersion; 4,000 rpm; time interval, 60 s; temperature, 25°C; and the number of scanning times, 255.

### Microstructure Analysis

The microstructure of emulsion samples was observed by confocal laser microscopy (FV1200, Olympus, Japan) after emulsions were diluted 4 times. In this experiment, samples were mixed with Nile Red (λex: 488 nm) to stain oil phase (RBO) and with Nile Blue A (λex: 640 nm) to stain proteins. Microstructure pictures were taken using a 10X eyepiece with a 60X objective lens (oil immersion). They were magnified 16 times by the software of the instrument (FV10i-Oil, Olympus, Japan).

### Shear Rheological Properties

The shear rheological properties were measured by a dynamic shear rheometer (MCR 301, WESP, Graz, Austria). It was equipped with cone-plate geometry (CP50-1). SPI-stabilized RBO emulsions with different concentrations of CMC were loaded into the rheometer measurement platform and equilibrated at 25°C for 2 min before making measurements. Shear stress and viscosity profiles were then performed in the range of 2–200 s^−1^.

### Rice Bran Oil Oxidation Measurements

Rice bran oil oxidative stability was determined by storing SPI-stabilized RBO emulsions with 0.5 wt% CMC in test tubes in the dark at 37°C for 30 days. Lipid hydroperoxides and thiobarbituric acid reactive substances (TBARSs) were determined according to the method of Long et al. (22). In short, hydroperoxide and TBARS were extracted using isooctane/isopropanol (3:1, v/v) mixture and TBA reagent, respectively. The absorbance of hydroperoxides was measured at 510 nm and the absorbance of TBARS was measured at 532 nm using a UV-vis scanning spectrophotometer, respectively (Shimadzu UVmini-1240, Kyoto, Japan). The cumene hydroperoxide calibration curve was used to determine hydroperoxide concentrations. The standard curve made for 1,1,3,3-tetra ethoxy propane was used to determine TBARS concentrations.

### *In-vitro* Digestion

Soy protein isolate-stabilized RBO emulsions with and without 0.5 wt% CMC were passed through an *in-vitro* digestion model. The simulated gastrointestinal tract model followed the method which involved mouth, stomach, and small intestine phases (23).

Simulated mouth phase: 3 g mucin was dissolved in 100 ml ultrapure water as the simulated saliva solution, and then adjusting the pH to 6.8. Initial emulsion samples were diluted in 1 mM, pH 3.5 phosphate buffer at a ratio of 1:5 (v/v) and then were mixed with simulated saliva solution at the ratio of 1:1 (v/v). The mixture was continuously shaken at 100 rpm for 10 min in a 37°C water bath.

Simulated stomach phase: the simulated gastric juice was prepared by dissolving 0.32 g pepsin, 0.2 g NaCl, and 0.7 ml HCl (12.0 M) in a volumetric flask (100 ml) with ultrapure water. Simulated stomach solution was then added to the saliva sample from the previous experiment at the ratio of 1:1 (v/v) and the mixture (pH 2.5) was stirred for 2 h (37°C, 100 rpm).

Simulated small intestine: 3 ml simulated intestinal fluid, 7 ml bile salt solution (53.6 mg/ml), 5 ml freshly prepared lipase solution (24 mg/ml) were successively added to a simulated stomach sample (60 g), respectively. The automatic titration unit (AT710S, KEM, Japan) was used to monitor the pH and maintain it at pH 7.0 by titrating 0.2 M NaOH solution into the reaction vessel for 2 h at 37°C.

#### Free Fatty Acid Release

The rate of free fatty acids (FFAs) released was calculated using the following formula:


$$\begin{array}{l} FFA\left(\%\right)=\frac{\left(V_{NaOH}\times m_{NaOH}\times M_{Lipid}\right)}{W_{Lipid}\times 2}\times 100\% \end{array}$$

Here *V*_NaOH_ is the volume of NaOH required to neutralize the produced FFA (in L), *m*_NaOH_ is the molarity of the added NaOH solution (0.2 M), *W*_Lipid_ is the total weight of lipid initially present in the reaction vessel (0.2 g), and *M*_Lipid_ is the mean relative molecular weight of RBO (273.64 g/mol).

#### Microstructure During *in-vitro* Digestion

The microstructure of SPI-stabilized emulsions with different concentrations of CMC was observed by confocal laser microscopy (FV1200, Olympus, Japan) after passing through an *in-vitro* digestion. All the microstructure pictures were taken using a 10X eyepiece with a 60X objective lens (oil immersion). The images were magnified 16 times by the software of confocal laser microscopy (FV10i-Oil, Olympus, Japan).

### Statistical Analysis

All the experiments were done in triplicate using freshly prepared RBO emulsions. Results are reported as the calculated mean and SD of replicates. Data were analyzed of variance (ANOVA) using the software package Origin 8.5.

## Results and Discussion

### Effect of the CMC Concentration on the Physical Stability of SPI-RBO Emulsions

#### Zeta Potential and Particle Size

**Figure 1:**
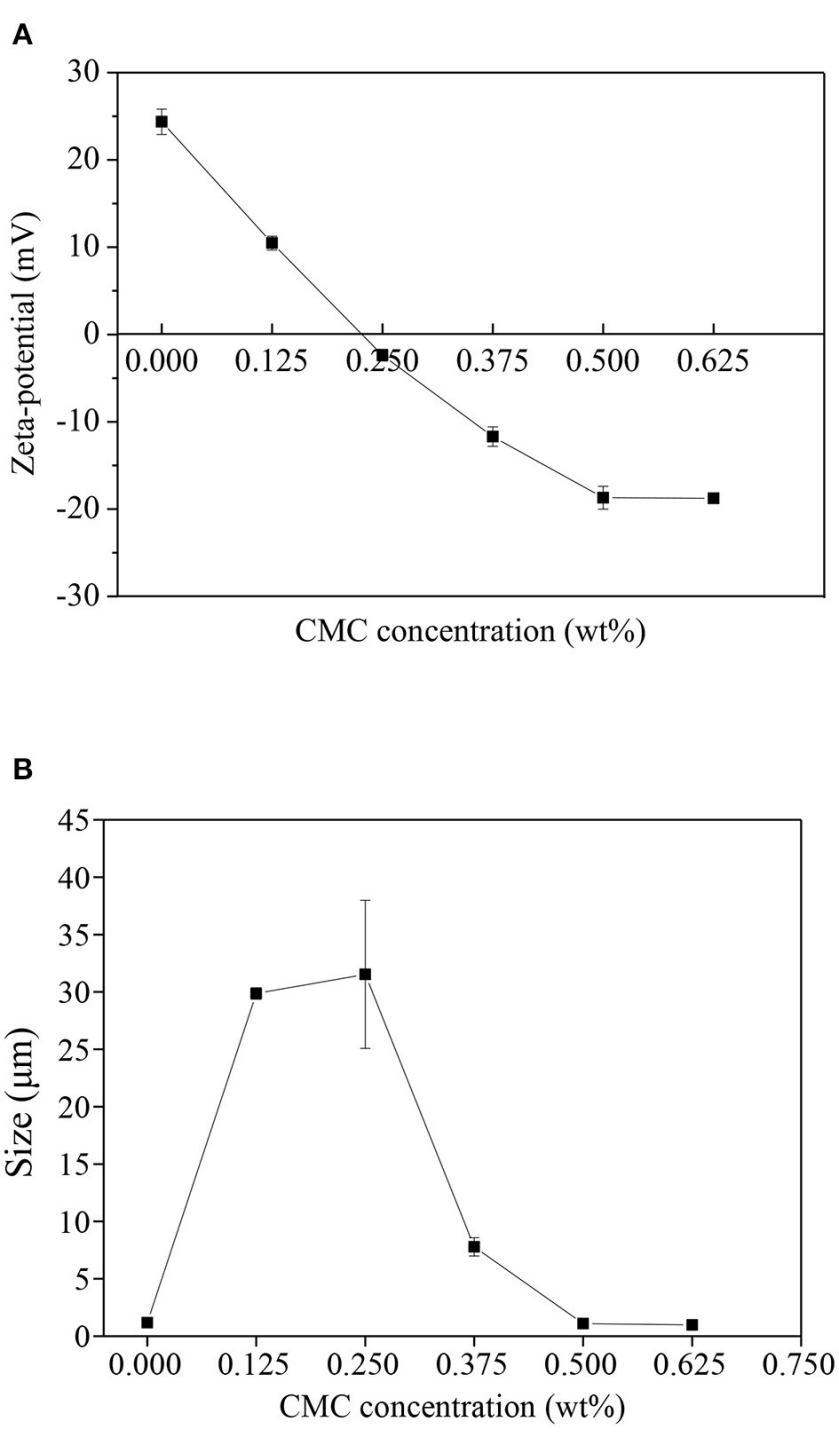
Effect of the different concentrations of carboxymethyl cellulose (CMC) on the zeta potential **(A,B)** particle size of SPI (1 wt%) stabilized rice bran oil (RBO) (6.67 wt%) emulsions at pH 3.5, 25°C. Error bars are SDs of mean values.

Zeta potential represents the stability of emulsion by reflecting the electrostatic interaction between droplets (24). The zeta potential of SPI-RBO emulsions stabilized with different concentrations of CMC at pH 3.5 was shown in Figure 1A. It showed that the net charge on the SPI-coated RBO droplets without CMC was around 24.4 ± 1.5 mV. The isoelectric point of SPI is about pH 4.5 (25), hence the SPI-coated RBO droplets were positively charged at pH 3.5, the electrical charge on the RBO droplets became less positive with the increase of CMC content, which indicated that the negatively charged CMC molecules absorbed to the surface of the positively charged RBO emulsion droplets forming SPI-CMC membranes (26). As an anionic polysaccharide, CMC contains a large number of negatively charged carboxyl groups, which can be combined with positively charged groups in 7S-β subunit and 11S-B polypeptide chains of SPI, such as amino groups, through electrostatic attraction (27). A small amount of CMC with 0.125 and 0.25 wt% decreased the zeta potential values of RBO emulsions to almost zero. It indicated that CMC at a lower concentration might bind to one or more RBO droplets and was not enough to form strong electrostatic repulsion interaction between droplets. At high contents of CMC (0.375–0.625 wt%), the emulsion droplets became negatively charged and finally the charge reached a plateau at −18.7 ± 1.3 mV when CMC concentration was 0.5 wt%, indicating that the droplets had become saturated with CMC or the increase of electrostatic repulsion force prevents the further adsorption of CMC (26). The ability of charged CMC to be adsorbed to the surface of charged emulsion droplets has been well evaluated (28).

The droplet size of SPI-RBO emulsions stabilized with different concentrations of CMC at pH 3.5 was shown in Figure 1B. It was shown that the droplet size of the SPI-RBO emulsion was about 1.2 μm. The smaller particle size may be due to the positive charge of the particle, which generates strong electrostatic repulsion. At lower CMC contents (0.125 and 0.25 wt%), there were significant increases in size (29.9 and 31.5 μm). This phenomenon may be attributed to the fact that there were insufficient CMC molecules present to completely cover SPI-coated oil droplet surfaces. As a result, CMC could bind to the surfaces of more than one cationic oil droplet leading to charge neutralization and bridging flocculation. With the further addition of CMC more than 0.25 wt%, there was a large decrease in the droplet size of RBO emulsion. When CMC content was more than 0.5 wt%, the droplet size of RBO emulsion reached a plateau and showed 1.0 μm. It indicated the interaction of CMC with SPI-coated RBO droplets and corresponded to the droplet charge given in Figure 1A. With the increase of CMC content, it would be enough to cover the RBO droplet's surface and form a thick interfacial layer. It was by other reports that CMC can interact with proteins by electrostatic interactions to form complexes (29). Therefore, the increased strong steric resistance and electrostatic repulsion between droplets can inhibit flocculation and reduce the particle size of droplets.

#### Physical Stability

**Figure 2:**
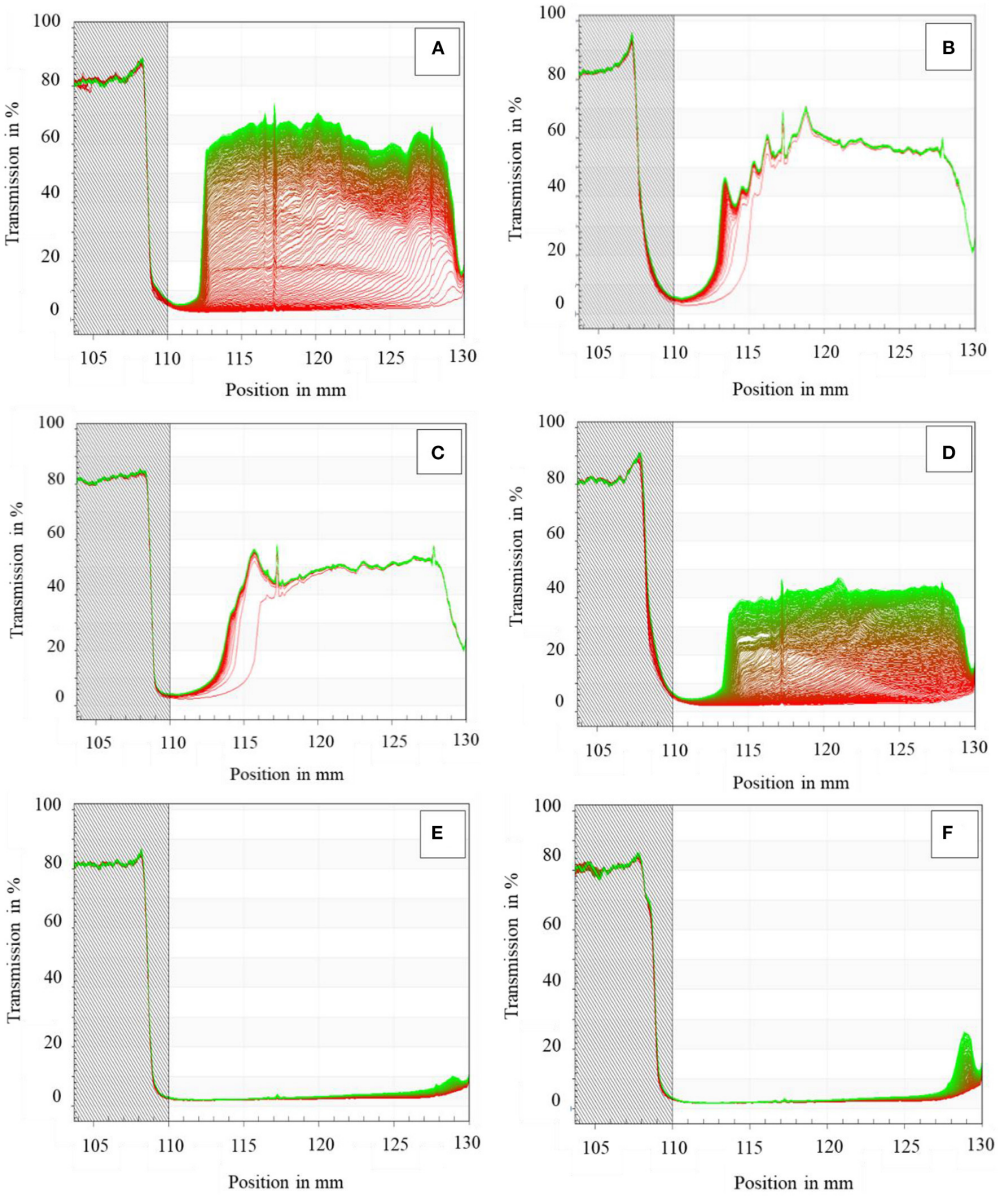
Physical stability tested by LUMiSizer (original transmission) of the SPI-stabilized rice bran oil emulsions with different concentrations of CMC at pH 3.5, 25°C [**(A)** 0 wt% CMC; **(B)** 0.125 wt% CMC; **(C)** 0.25 wt% CMC; **(D)** 0.375 wt% CMC; **(E)** 0.5 wt% CMC; and **(F)** 0.625 wt% CMC].

According to our previous study (30), the more change of the transmission with the centrifugation is, the less stable the emulsion is. Figures 2A–F exhibits the original transmission profiles of SPI-coated RBO emulsions stabilized with different concentrations of CMC ranging from 0 to 0.625 wt%. The positions in the transmission profiles at about 105 and 130 mm corresponded to the filling height and cell bottom, respectively. It was obvious that with increasing the CMC content from 0 to 0.625 wt%, the last integral transmissions were lower along the sample length, indicating that emulsions were more stable. It can be seen in Figures 2A–F that a sharp front moved toward the cell top during centrifugation, which means that RBO droplets of all the emulsions were moving as a collective (zone creaming). Figure 2A shows the original transmission of sample position of emulsion without CMC, presenting the obvious creaming compared with other emulsions. It was also found that the first profiles of emulsions stabilized with 0.125 wt% CMC (Figure 2B) and 0.25 wt% CMC (Figure 2C) exhibited higher transmission along the sample length, which might be due to the flocculation of large particles in emulsions during centrifugation. As the amount of CMC increased from 0.375 wt% (Figure 2D) to 0.5 wt% (Figure 2E), the last profile of all emulsions was lower than that without CMC along the sample length, which indicated that the stability of emulsion increases with the increase of CMC addition, which may be due to enhanced steric hindrance and droplet network in RBO emulsion. The transmission peak of emulsion stabilized with 0.625 wt% CMC (Figure 2F) was higher and wider than that with 0.5 wt% CMC, indicating that the former was less stable than the latter. This might be depletion flocculation caused by the addition of excessive CMC (31).

#### Microstructure Analysis

**Figure 3:**
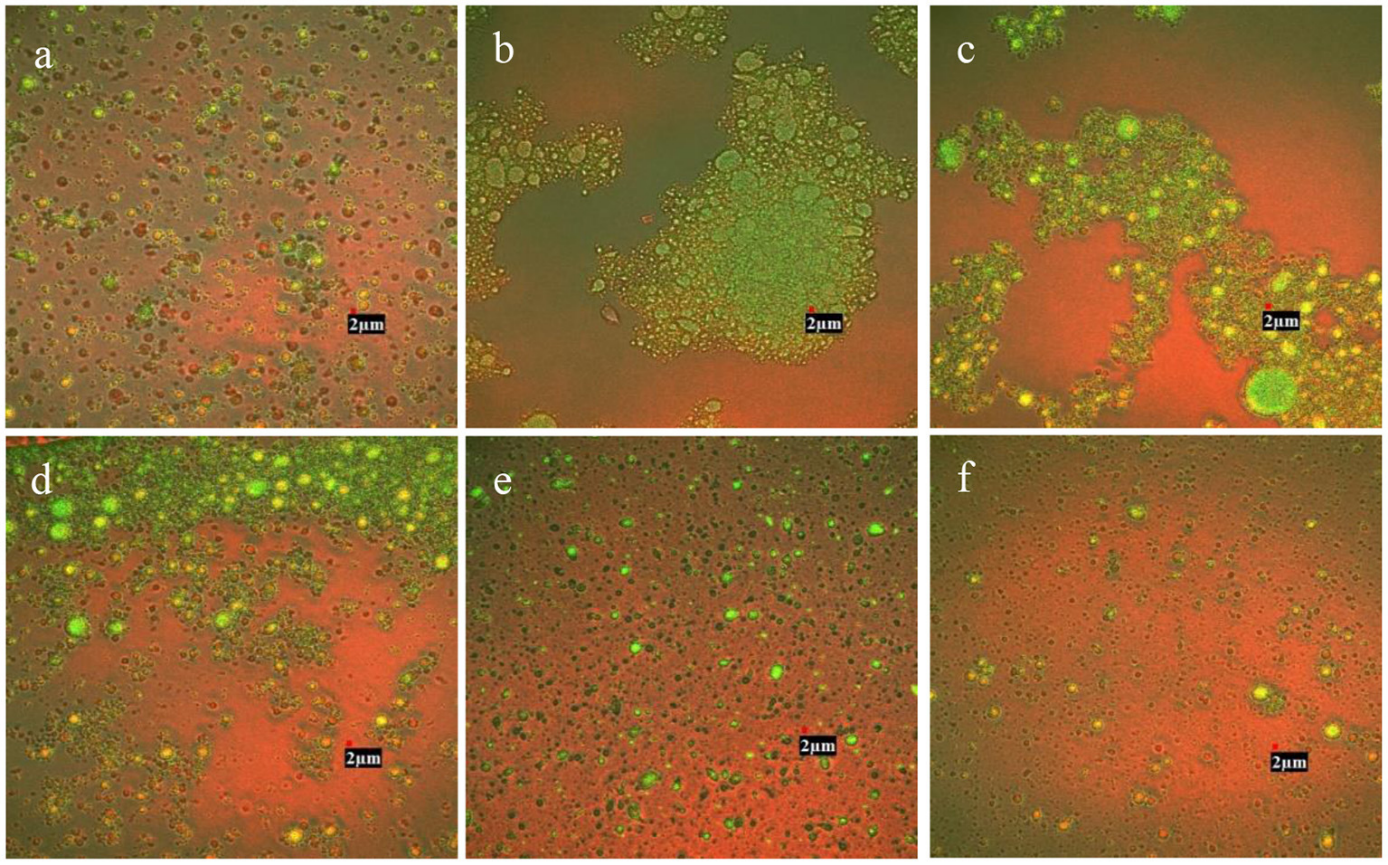
The confocal laser scanning microscopy images of the SPI-stabilized rice bran oil emulsions with different concentrations of CMC at pH 3.5, 25°C [**(A)** 0 wt% CMC; **(B)** 0.125 wt% CMC; **(C)** 0.25 wt% CMC; **(D)** 0.375 wt% CMC; **(E)** 0.5 wt% CMC; and **(F)** 0.625 wt% CMC]. All the photos were magnified at 30,000X.

Confocal laser scanning microscopy analysis demonstrated the microstructure of RBO emulsions stabilized with different concentrations of CMC (Figure 3). The green area represented RBO phase enrichment and the red area presented the SPI enrichment phase region. It was found that emulsion stabilized with 0.125 wt% CMC and 0.25 wt% CMC appeared extensive droplet flocculation compared with RBO emulsion without CMC. It might be due to the net charges of the particles in these two emulsions approaching zero, resulting in weakening the electrostatic repulsion. On the other hand, the concentrations of CMC were relatively low and the space resistance was relatively small. Combined with the above reasons, aggregation was more likely to occur between particles. The result was also consistent with the conclusion of particle size measurement (Figure 1A). As can be seen from Figure 3D, the particles in the emulsion with 0.375 wt% CMC exhibited slight flocculation, which might be the consequence of the enhancement of electrostatic repulsion and steric resistance. In the emulsion containing 0.5 or 0.625 wt% CMC, the droplet showed a relatively uniform distribution compared with the emulsion containing 0.125 wt% CMC, and the presence of CMC did not lead to flocculation in the emulsion. This might be because the particles in emulsions were negatively charged in this circumstance, increasing electrostatic repulsion between the particles. At the same time, the increase of CMC concentration also increased the spatial resistance between particles. Finally, the particles were uniformly dispersed and not easy to aggregate. Ultimately this indicated that RBO emulsions stabilized with 0.5 wt% CMC and 0.625 wt% CMC were more stable than others, which was in accordance with the result of physical stability and droplet size of RBO emulsions stabilized with different concentrations of CMC.

### Shear Rheological Properties

**Figure 4:**
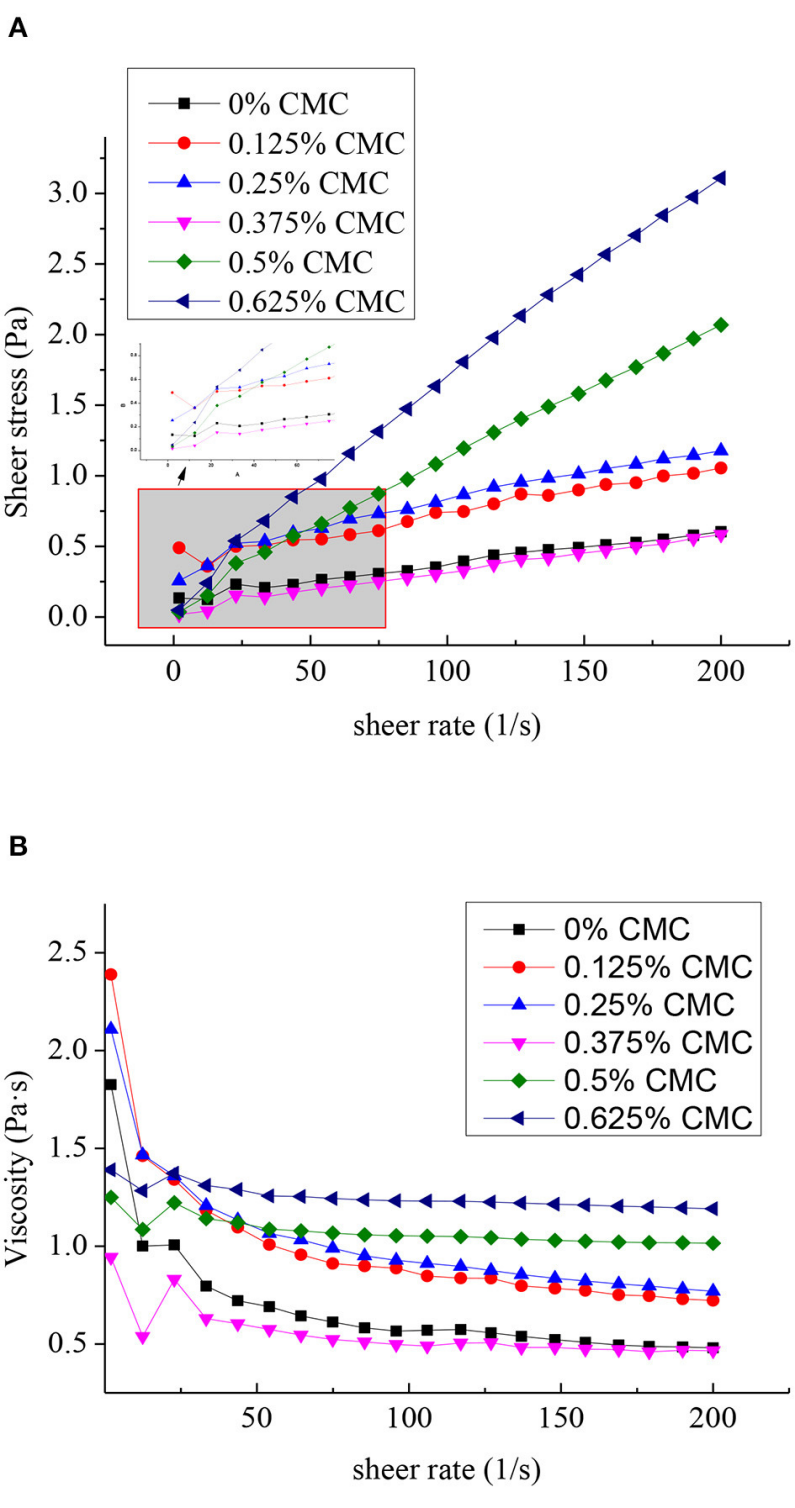
Flow curves **(A)** and the apparent viscosity **(B)** of the SPI-stabilized rice bran oil emulsions with different concentrations of CMC (shear rate ranging from 2 to 200 s^−1^ at pH 3.5, 25°C).

Flow curves and the apparent viscosity of SPI-stabilized RBO emulsions with different concentrations of CMC with the shear rate in the range of 2 to 200 s^−1^ were characterized, as shown in Figure 4. In the RBO emulsions stabilized with different concentrations of CMC, significantly higher shear stress was observed with increasing concentrations of CMC, except for the RBO emulsion stabilized with 0.375 wt% CMC (Figure 4A). This might be because the additional amount of CMC just reached saturation, that is, each CMC molecule was wrapped on the surface of SPI-RBO particles under the action of electrostatic attraction, and the newly formed particles had a smooth surface and were similar in structure to RBO emulsion without CMC. The result was insistent with the particle size and zeta potential results.

The effect of different concentrations of CMC on the apparent viscosity of RBO emulsions was exhibited in Figure 4B. It showed that the apparent viscosity of all RBO emulsions decreased appreciably with the shear rate increased, indicating that the addition of CMC promoted SPI-RBO emulsions to become pseudoplastic fluid. This shear-thinning behavior could be that the internal structure of the RBO emulsion opens with increasing the shear rate, and the molecules were linearly aligned in the direction of the shear rate (32). When the shear rate was greater, the viscosity curve tended to be flat with increasing shear rate, indicating that it was close to Newtonian fluid. The viscosity of SPI-RBO emulsions with 0.5 wt% CMC was significantly higher than that of SPI-RBO emulsion alone, which may be due to the larger particle size, forming a specific spatial structure, thus increasing the viscosity value. It was worthy to mention that the viscosity of RBO emulsion stabilized with 0.375 wt% CMC was close to that of RBO emulsion without CMC. The structural similarity of the particles in the two RBO emulsions might be the cause of this phenomenon (33, 34).

### Chemical Stability

**Figure 5:**
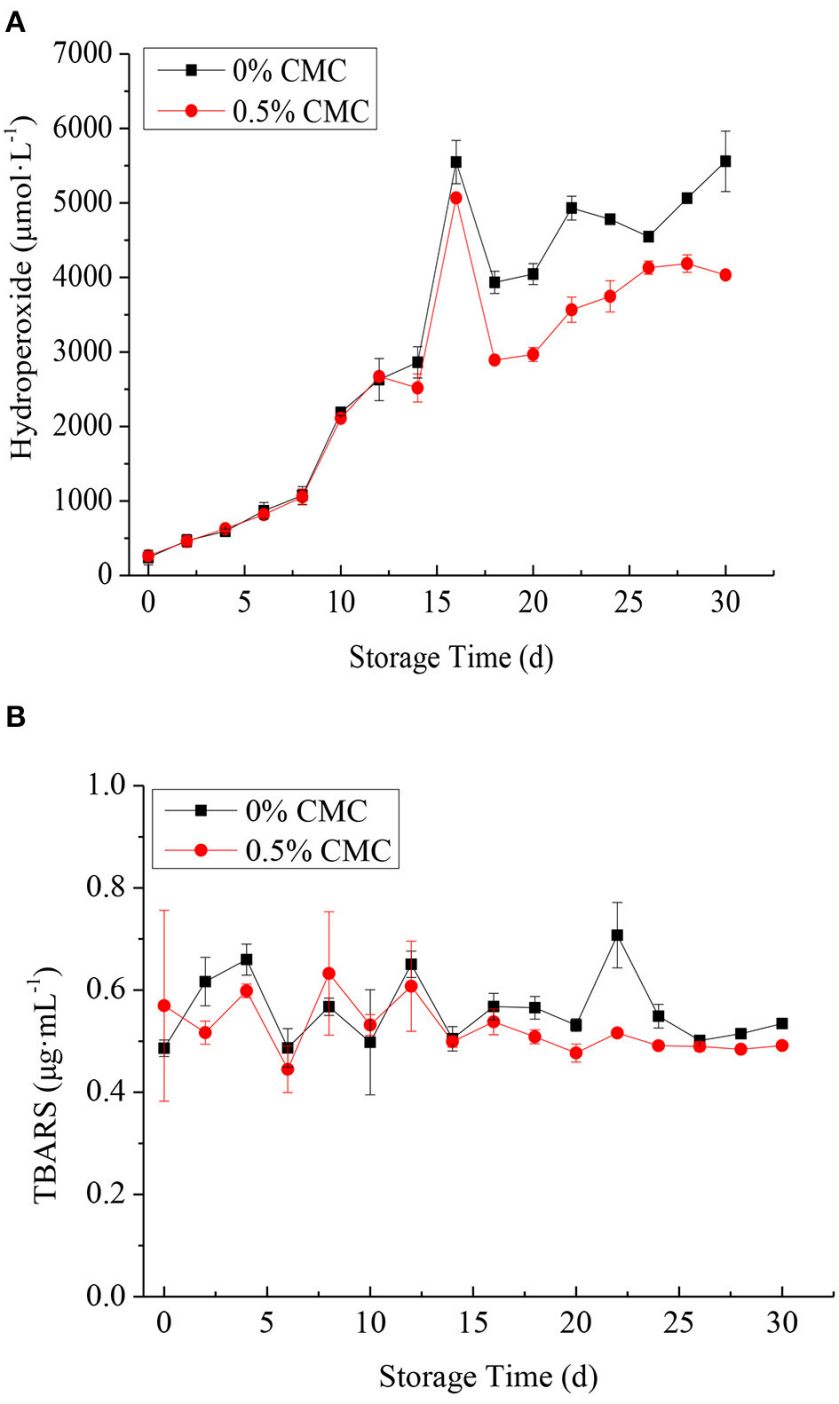
The formation of lipid hydroperoxides (μmol/l emulsion) **(A)** and thiobarbituric acid reactive substances (TBARS) (μg/ml emulsion) **(B)** in SPI-stabilized rice bran oil emulsions with 0.5 wt% CMC during the incubation at 37°C. Data points represent means (*n* = 3) ± SDs.

To measure the differences in oxidative stability between SPI-RBO emulsions without and with 0.5 wt% CMC, these emulsions were stored at 37°C in the dark for 30 d to accelerate oxidation rates. Figure 5A shows the content of lipid hydroperoxides in SPI-RBO emulsions without and with 0.5 wt% CMC during the incubation. SPI-RBO emulsions showed a more dramatic increase in lipid hydroperoxide formation than that stabilized with 0.5 wt% CMC. Lipid hydroperoxides increased slightly from day 0 to 8 in the SPI-RBO emulsions with and without 0.5 wt% CMC. After that, fast increases in lipid hydroperoxide formation were found, with the largest increase in lipid hydroperoxide at day 16. The concentrations of lipid hydroperoxides in the SPI-RBO stabilized without and with 0.5 wt% CMC were 5,548.9 and 5,066.7 μmol lipid hydroperoxides/l, respectively. After that, lipid hydroperoxides of both the RBO emulsions decreased obviously. It could be due to the oxidation of lipid hydroperoxides into the secondary lipid oxidation products.

In addition, secondary lipid oxidation product TBARS was also determined for the monitoring of oxidative stability of SPI-RBO emulsions during storage. The results of TBARS concerning SPI-RBO emulsions without and with 0.5 wt% CMC are given in Figure 5B. It was found that the content of TBARS in the SPI-RBO emulsion was more than that in the emulsion stabilized with 0.5 wt% CMC after storing 30 days, indicating that CMC could reduce the accumulation of TBARS. No obvious change in the content of TBARS (≈ 0.6 μg/ml) was observed in both RBO emulsions after storage of 16 d. However, the TBARS of RBO emulsion was increased obviously at day 22 in RBO emulsion without CMC. The TBARS concentration in RBO emulsion without CMC was 0.71 μg/ml TBARS. It shows that in the absence of CMC, more primary oxidation products decompose to form secondary oxidation products. After that, the RBO oxidation product of emulsions tended to decrease, which might be due to the additional by-products. Overall, the RBO emulsion stabilized with 0.5 wt% CMC was more effective in inhibiting the lipid oxidation compared to the single SPI-coated RBO emulsion, which might have two possible explanations. First, the carboxyl group in CMC interacts with the amino group in SPI to form SPI-CMC complex, which can be functioned as an emulsifier to form a layer of hydrated interfacial film on the surface of RBO, which improves the surface viscosity and density of droplets, thus improving the stability of emulsions (26, 35). Second, the steric hindrance effect produced by macromolecules reduces the contact between oxygen, transition metals, and RBO, thus reducing the release of free radicals and delaying the oxidative decomposition of RBO. In general, the composite membrane structure formed by SPI and CMC could slow down oil oxidation. It was consistent with the study of Ma et al. (36).

### *In-vitro* Digestion

**Figure 6:**
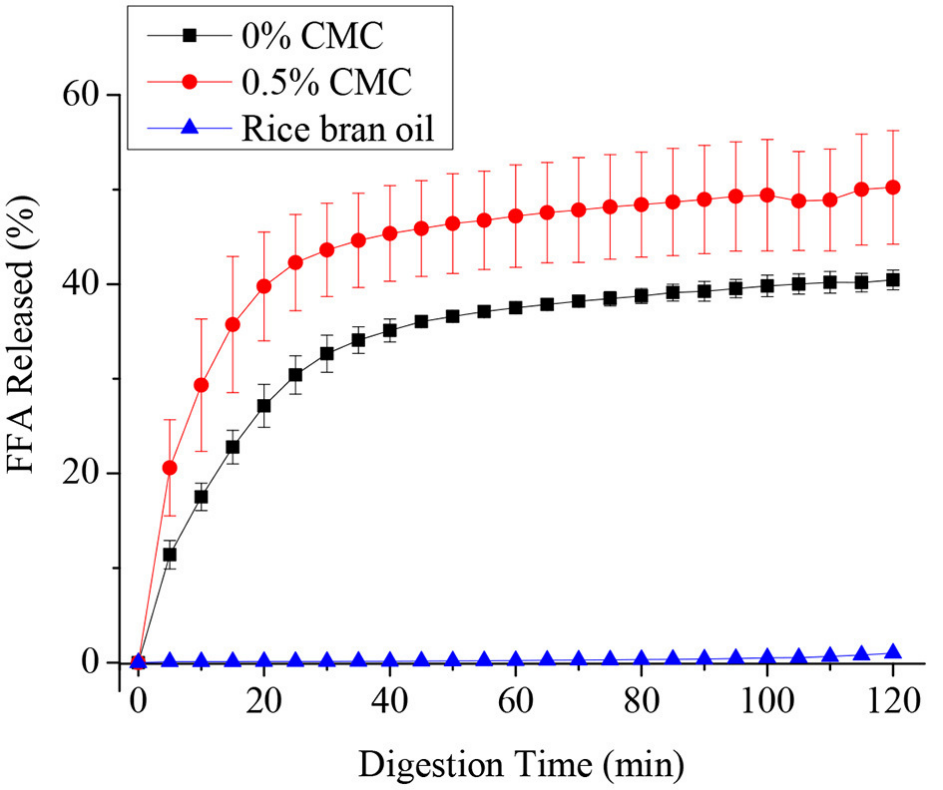
Free fatty acids (FFAs) released from SPI-stabilized rice bran oil emulsions with 0.5 wt% CMC and pure rice bran oil after passing through an *in-vitro* digestion model (oral, gastric, and intestinal phases).

Figure 6 shows the FFA release profiles of RBO emulsions stabilized with and without 0.5 wt% CMC during intestinal digestion. In the first 30 min, the FFA release rate of the two emulsions was very fast and reached a plateau at about 40 min. The reason for this phenomenon might be that with the progress of enzymatic hydrolysis reaction, the concentration of substrate decreased and the reaction site was gradually saturated by lipase. When the concentration of digestive products increased, its competitive inhibition became stronger, or enzyme activity decreased.

It can be seen that the RBO emulsions without CMC have a higher FFA release rate than pure rice bran oil, their final percentage of FFA release was around 30 and 1%, respectively. While around 50% FFA was released after digestion in the SPI-RBO emulsions stabilized with 0.5 wt% CMC. This result indicated that RBO digestion was appreciably improved by the emulsification of SPI. Emulsion stabilized by CMC could further accelerate the release of fatty acid and improve the digestibility of RBO. Compared with a single aqueous phase or an organic phase, lipase can play a better catalytic role on the oil-water interface, because the aqueous phase can disperse enzyme molecules and maintain the dominant conformation of enzyme protein, while the organic phase can open the lid structure on the enzyme active center, allowing substrate molecules to enter the enzyme active center, thus activating the enzyme (37). Hydrophilic groups and lipophilic groups in SPI can accelerate the combination of lipase and lipid droplets to degrade fat. Therefore, the free fatty acid release rate of SPI-RBO emulsion was higher than that of pure RBO, while CMC contains a large number of hydrophilic groups such as hydroxyl and carboxyl, which can optimize the catalysis of lipase and accelerate the release of FFA (38, 39).

### Structural Changes During *in-vitro* Digestion

**Figure 7:**
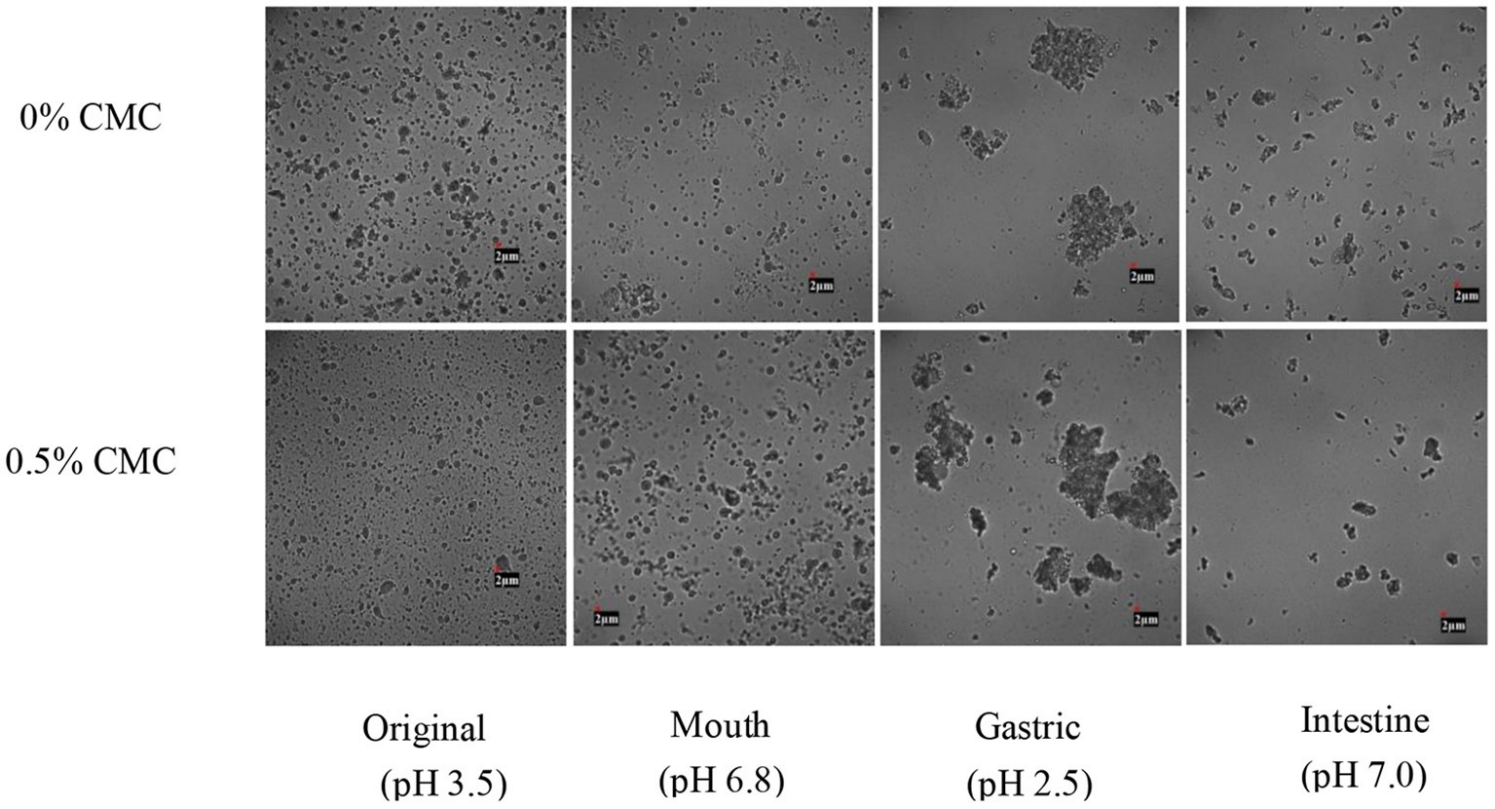
The microstructures of different stages of SPI-stabilized rice bran oil emulsions with 0.5 wt% CMC after passing through an *in-vitro* digestion model (original, oral, gastric, and intestinal phases).

Figure 7 shows the microstructure of SPI-RBO emulsions with and without 0.5 wt% CMC during different stages of *in-vitro* digestion. First, there was no significant difference occurred in the simulated oral phase between both emulsions. It can be seen that mucin had no obvious effect on inducing emulsifier bridging or depletion flocculation in the simulated oral phase, which might be due to the short reaction time and mild reaction conditions, such as moderate pH (40, 41).

However, after the stomach, the droplets began to show obvious flocculation and aggregation. It could be attributed to the fact that the pepsin could hydrolyze SPI to destroy the structure of the original emulsifier, and the oil phase was driven by hydrophobic action to flocculate and deposit again. In addition, due to the strong acidity of gastric juice and the high ionic strength environment, interfacial layer hydrolysis and lower electrostatic repulsion force would increase the possibility of droplet flocculation (42–44).

After digestion in the simulated small intestine phase, droplets in both RBO emulsions were less compared with the original ones, indicating that some droplets were degraded and utilized. This was because lipase on the surface of the oil had a digestive effect on emulsified lipid. The lipase adhered to the surface of the lipid droplet and degraded triacylglycerol into free amino acids and triacylglycerol, thus changing the interfacial properties of the emulsion (45). SPI-RBO emulsion without CMC still contained relatively large droplets than that with 0.5 wt% CMC, which indicated that RBO emulsion with 0.5 wt% CMC had undergone more thorough degradation. The reason might be that the addition of CMC, especially hydrophilic groups, such as hydroxyl and carboxyl, accelerated the adsorption rate of lipase, thus making fatty acids easier to be released (46–48).

## Conclusion

This study had characterized the impact of CMC on the physicochemical stability, rheological property, and *in-vitro* digestion of SPI-RBO emulsions. It is concluded that 0.5 wt% negatively charged CMC can be uniformly coated on a positively charged SPI-RBO droplet at pH 3.5, and the emulsion exhibited a smaller particle size, uniform distribution, and improved physical stability. The stable structure of the emulsion is formed by electrostatic interaction and steric hindrance between CMC and SPI on the droplet surface. Compared with SPI-RBO emulsion, adding CMC can effectively reduce the oxidation products produced during long-term storage. In addition, *in-vitro* digestion experiments showed that the FFA release rate of CMC-stabilized SPI-RBO emulsion added with 0.5 wt% was significantly higher than that of pure RBO and SPI-RBO emulsion, indicating that CMC had the potential to improve RBO bioaccessibility and was beneficial to the use of bioactive substances in RBO. To sum up, the use of CMC-stabilized SPI-RBO emulsion can improve the theoretical basis for the development of functional factors delivery system in food production.

## Data Availability Statement

The original contributions presented in the study are included in the article/Supplementary Material, further inquiries can be directed to the corresponding author.

## Author Contributions

WZ: methodology, formal analysis, writing–review, and editing. JH: methodology and review. YY: methodology, investigation, and validation. DX: conceptualization, writing–review and editing, project administration and supervision, and funding acquisition. All authors contributed to the article and approved the submitted version.

## Funding

This study was funded by the National Natural Science Foundation of China (32072216), the Graduate Innovation Project of School of Food and Health in 2021 of Beijing Technology and Business University. CIFST - Abbott Foundation of Food Nutrition and Safety (No. 2021-F04).

## Conflict of Interest

The authors declare that the research was conducted in the absence of any commercial or financial relationships that could be construed as a potential conflict of interest.

## Publisher's Note

All claims expressed in this article are solely those of the authors and do not necessarily represent those of their affiliated organizations, or those of the publisher, the editors and the reviewers. Any product that may be evaluated in this article, or claim that may be made by its manufacturer, is not guaranteed or endorsed by the publisher.
