# Supplementary figures and images for: Influence of Carboxymethyl Cellulose on the Stability, Rheological Property, and in-vitro Digestion of Soy Protein Isolate (SPI)-Stabilized Rice Bran Oil Emulsion
Source: Front Nutr. 2022 Apr 11;9:878725. doi: 10.3389/fnut.2022.878725 (PMC9037688; doi:10.3389/fnut.2022.878725)

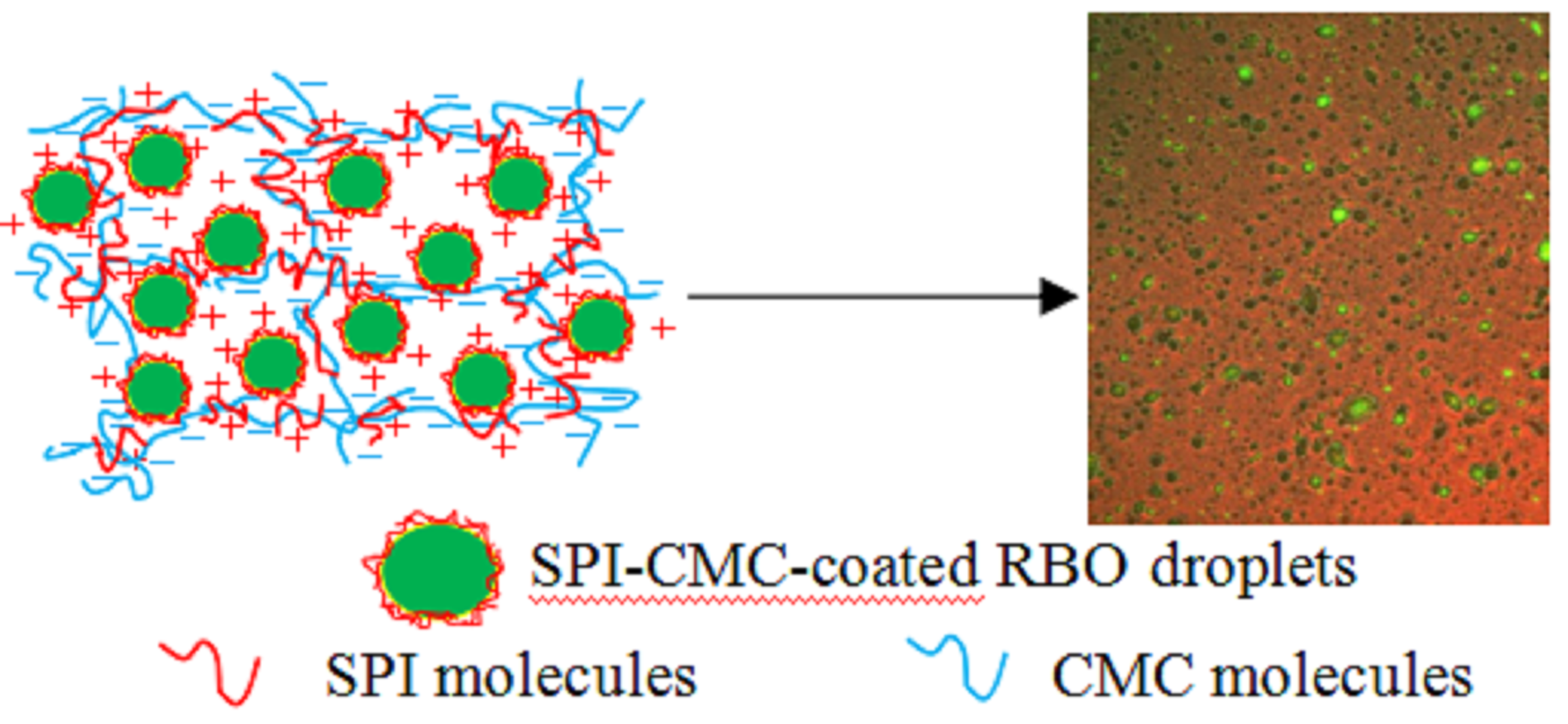

Supplement: Supplementary file 1 [file Image_1.TIF]
